# A phase I (tumour site-specific) study of carboplatin and temozolomide in patients with advanced melanoma

**DOI:** 10.1038/sj.bjc.6601414

**Published:** 2003-11-11

**Authors:** S J Strauss, M Marples, M P Napier, T Meyer, J Boxall, G J S Rustin

**Affiliations:** 1 Mount Vernon Cancer Centre, Rickmansworth Road, Northwood, Middlesex HA6 2RN, UK

**Keywords:** advanced melanoma, temozolomide, carboplatin, phase I study

## Abstract

Temozolomide is an oral alkylating agent that readily crosses the blood–brain barrier and has activity in patients with advanced melanoma. Carboplatin is a convenient outpatient treatment that also has activity in patients with melanoma. The purpose of this study was to assess the safety of a combination of temozolomide and carboplatin, and provide preliminary evidence of efficacy. In all, 30 patients were treated in two stages. In stage 1, patients received temozolomide 750 mg m^−2^, with escalating doses of carboplatin AUC 3–6. In stage 2, patients received temozolomide 1000 mg m^−2^, with increasing doses of carboplatin until dose-limiting toxicity (DLT) was experienced. In stage 1, 12 patients received 33 cycles of treatment. No grade 3/4 haematological toxicity was experienced up to carboplatin AUC 6. In stage 2, 18 patients received 55 cycles of treatment. The DLT was haematological with grade 4 myelosuppression seen with carboplatin AUC 5. In all, 11 patients were treated with carboplatin AUC 4 to gain further information on toxicity. Myelosuppression remained significant and common with grade 4 thrombocytopenia experienced in 50% of cycles. Two of 28 patients (7%) assessable for efficacy achieved a partial response. None of the 11 patients with brain metastases responded to treatment. The addition of carboplatin to temozolomide 1000 mg m^−2^ significantly adds to toxicity with frequent grade 3/4 myelosuppression. Preliminary information on efficacy demonstrates that it is unlikely that the combination can be given in doses sufficient to improve on the efficacy of temozolomide alone.

Patients with advanced melanoma have a poor prognosis, despite the use of chemotherapeutic and biological agents. Response rates to cytotoxic agents are generally poor, although a number of agents have activity. The most active agent is thought to be dacarbazine (DTIC) with a response rate of 11–25%; however, responses are not durable, and the median survival for this group of patients is 4.5–6 months (Lee *et al*, 1995). Combination chemotherapy has not added significantly to outcome. The Dartmouth regimen, comprising DTIC, cisplatin, carmustine and tamoxifen initially demonstrated a response rate of 55% in 20 patients treated (Del Prete *et al*, 1984). Subsequent single-centre phase II trials confirmed response rates of 40–50% (McClay *et al*, 1992; Richards *et al*, 1992), but this was not substantiated in phase III randomised trials. A randomised trial examining the contribution of tamoxifen to the regimen showed response rates of 20–30%, but no benefit from its addition (Rusthoven *et al*, 1996). Recently, single-agent DTIC has been compared to the Dartmouth regimen in a randomised-controlled trial, with no difference in survival between the two treatment arms being seen. There was a nonsignificant increase in the response rate with the combination arm. Toxicity, particularly relating to myelosuppression, was significantly increased with the Dartmouth regimen (Chapman *et al*, 1999). As chemotherapy for this group of patients is palliative, improvements in tolerability and ease of administration are worth investigating.

Temozolomide is an oral alkylating agent of the imidazotetrazine group, with a broad spectrum of antitumour activity. Its active metabolite (MTIC) is the same as that of dacarbazine (Tsang *et al*, 1991). It was developed as a potential alternative to DTIC and was found to have >90% oral bioavailability, and extensive tissue distribution, including penetration of the blood–brain barrier and the cerebrospinal fluid (CSF) (Stevens *et al*, 1987). This is particularly advantageous in patients with metastatic melanoma, as up to 75% of patients develop brain metastases (Dreiling *et al*, 1996). The initial phase I study demonstrated that temozolomide is well tolerated with a maximum tolerated dose (MTD) of 1000 mg m^−2^ (given as 200 mg m^−2^ once a day over 5 consecutive days). The dose-limiting toxicity (DLT) is myelosuppression with a nadir at 21 days and recovery usually by 28 days. Mild to moderate emesis occurs in approximately a third of patients, but is easily controlled with standard antiemetics. Prolonged or severe emesis is unusual (Newlands *et al*, 1992). A phase II trial of temozolomide conducted in patients with metastatic melanoma demonstrated an objective response rate of 21%, with three complete responses and nine partial responses in 56 evaluable patients (Bleehen *et al*, 1995). In a randomised phase III trial of 305 patients, temozolomide was compared to DTIC and demonstrated equal efficacy with improved health-related quality of life (Middleton *et al*, 2000).

Platinum compounds have been evaluated in the treatment of metastatic melanoma, both alone and in combination. Single-agent cisplatin yields responses of over 10% (Al-Sarraf *et al*, 1982) and has also been used in combination with DTIC in phase II trials, showing responses of over 30% (Murren *et al*, 1991). The combination is better tolerated than other combinations including the Dartmouth regimen, and avoids the delayed myelosuppressive problems associated with carmustine. These results, however, have not been evaluated in any phase III trials. Carboplatin has similar single-agent activity to cisplatin, with a dose of 400 mg m^−2^ demonstrating a response rate of 16% in a phase II trial of 23 patients (Evans *et al*, 1987). Carboplatin is an attractive alternative to cisplatin because of the ease of administration and favourable side effect profile.

Temozolomide and carboplatin have not previously been used in combination, but is a potentially attractive regimen as carboplatin is administered as a short intravenous infusion, which can be given in an outpatient setting unlike cisplatin-containing therapies, and temozolomide is given orally. There is the potential for overlapping toxicities especially relating to myelosuppression, but the median time to nadir leucocyte and platelet count is different for the two drugs. Other toxicities are expected to be uncommon. The combination of temozolomide and carboplatin potentially offers patients with advanced melanoma an improvement in efficacy with a convenient regimen.

The primary aim of the trial was to treat cohorts of patients with advanced melanoma with escalating doses of carboplatin and temozolomide to establish the MTD and DLT of this combination. The trial was conducted in two stages, the first with temozolomide at a dose of 750 mg m^−2^, and the second at a dose of 1000mg m^−2^. We report on the principal toxicities of the combination of carboplatin and temozolomide and provide preliminary data of efficacy, in patients with bidimensionally measurable disease.

## PATIENTS AND METHODS

### Patient selection

Patients with histologically confirmed, unresectable or metastatic, malignant melanoma, with measurable or assessable lesions, were eligible for the study. Patients were required to be over 18 years, have a life expectancy of at least 3 months and have an Eastern Cooperative Oncology Group (ECOG) performance status of <2. No prior chemotherapy was allowed, but radiotherapy was permissible to nonindicator lesions completed at least 3 weeks prior to entry. Eligibility criteria included Hb >10 g dl^−1^, WBC >3 × 10^9^ l^−1^, platelets >100 × 10^9^ l^−1^, serum creatinine of <120 mmol l^−1^ or creatinine clearance of >50 ml min^−1^, bilirubin <30 mmol l^−1^, AST <3 × upper limit of normal (ULN) and alkaline phosphatase <3 × ULN. Written informed consent was obtained from all patients prior to entry. The study had local ethical and scientific committee approval.

### Pretreatment investigations

Blood investigations including full blood count, renal function and liver function were measured. Renal function was measured using ^51^Cr-EDTA clearance technique, and urinalysis was also performed. Radiological assessment of site/s of disease was carried out with computer tomography (CT), magnetic resonance imaging (MRI) or clinical photographic measurement, as appropriate. Chest radiograph and ECG were performed.

### Treatment schedule

In the first stage, patients in cohort 1 were given carboplatin AUC 3 intravenously on day 1, and temozolomide 150 mg m^−2^ administered orally for 5 days. Treatment was given at 28-day intervals for up to six cycles. In successive cohorts, doses of carboplatin were increased by AUC 1 until AUC 6 was reached. In stage 2, patients in the first cohort were given temozolomide 200 mg m^−2^ orally for 5 days and carboplatin AUC 3 intravenously on day 1, at 28-day intervals, up to six cycles. Carboplatin dose was escalated by AUC 1 in further cohorts until DLT was experienced. Patients who responded to treatment, or had stable disease on completion of six cycles of the combination were eligible for continuation of temozolomide alone for six further cycles of treatment in accordance with the regimen used in the phase III trial comparing temozolomide with DTIC, where up to 12 cycles of treatment were permitted (Middleton *et al*, 2000).

### Study design

Cohorts of three patients were treated at escalating doses of the study drugs. Patients were assessed for CALGB expanded Common Toxicity Criteria prior to each treatment cycle. At each dose level, the first patient was observed for 3 weeks or until recovery from acute toxicity (4 weeks for grade 4 toxicity) before further patients were entered at the same dose level. The DLT was defined as grade 3 or 4 haematological toxicity, grade 4 nonhaematological toxicity or grade 2 cardiac or pulmonary toxicity. Dose escalation was considered when at least three patients had been entered at a given dose, and two patients followed for 3 weeks or until all acute toxicities had resolved. If no patients in a cohort had unacceptable toxicity, dose escalation was undertaken. If one of three patients had unacceptable toxicity, then three further patients were treated at the same dose. The DLT level was reached when two of three patients experienced DLT. A minimum of 10 patients were to be treated at the dose level immediately below which DLT was experienced in an attempt to establish the MTD.

### Toxicity evaluation and dose modification for toxicity

Patients were evaluated for toxicity prior to each treatment cycle, and full blood count and renal and liver function tests were performed. Nadir full blood counts were measured on days 15 and 22 of each cycle. The ANC was required to be >1.5 × 10^9^ l^−1^ and the platelet count >100 × 10^9^ l^−1^ on day 28 for further treatment. The use of growth factors to maintain dose was not permitted. Dose modification was specified according to toxicity. Chemotherapy was delayed for up to 1 week without modification if ANC count on day 28 was 1–1.5 × 10^9^ l^−1^ or platelet count >50–<100 × 10^9^ l^−1^. If ANC was <1 × 10^9^ l^−1^ or platelet count <50 × 10^9^ l^−1^ on day 28, treatment was delayed until recovery and carboplatin dose reduced by AUC=1. Dose delays of >2 weeks constituted unacceptable toxicity and chemotherapy was withdrawn.

All nonhaematological toxicity >grade 1 was required to have resolved to grade 1 or baseline prior to repeat dosing. No dose adjustments were made for resolved grade 2 toxicity. In patients with grade 3 nonhaematological toxicity, the dose of carboplatin was reduced by AUC 1 for further cycles of treatment. Patients experiencing grade 4 nonhaematological toxicity, unless considered unrelated to study drugs, were withdrawn from the study.

### Response assessment

Prior to treatment, patients underwent clinical and radiological assessment of site/s of disease with radiographs, CT or MRI where appropriate. Patients could be assessed for response or progressive disease if they received one or more cycles of treatment, although radiological response was assessed after every two cycles of treatment. Patients could be assessed for progressive disease after one cycle of treatment if symptoms dictated. Tumour response was defined according to EORTC response criteria (Therasse *et al*, 2000). Further investigations were undertaken according to clinical evaluation.

## RESULTS

### Patient characteristics

In all, 30 patients with a median age of 54 years were treated between December 1998 and January 2001. Of these, 22 patients had multiple sites of metastases and 11 had brain metastases. Patient characteristics are listed in Table 1

**Table 1:** Patient characteristics

Total number of patients	30
*Sex*	
Male	16
Female	14
	
*Age* (years)	
Median	54
Range	40–81
	
*ECOG performance status*	
0	16
1	10
2	4
	
*Time to distant progression from diagnosis* (months)	
Median	35.4
Range	0–126
	
*Site of disease*	
Skin/soft tissue	14
Nodal disease	9
Lung	15
Liver	11
Brain	11
Bone	5
Visceral disease (nonhepatic)	4
Single site only	8

ECOG=Eastern Cooperative Oncology Group.

.

### Treatment

In total, 30 patients received 89 cycles of treatment (Table 2

**Table 2:** Dose escalations

**Carboplatin dose**	**No. assessable patients**	**No. courses**	**No. with DLT**
*Stage 1: Temozolomide dose 750 mg m*^−*2*^			
AUC 3	3	10	0
AUC 4	3	11	0
AUC 5	3	7	0
AUC 6	3	5	0
			
*Stage 2: Temozolomide dose 1000 mg m^−2^*			
AUC 3	3	13	0
AUC 4	11	35	8
AUC 5	4	8	2

DLT=dose limiting toxicity.AUC=Area under curve.

). In stage 1, at temozolomide 750 mg m^−2^, 12 patients received 33 cycles of treatment. The median number of courses per patient was two (range 1–6). One patient completed six cycles of treatment. No patients experienced DLT. In stage 2 (temozolomide 1000 mg m^−2^), 18 patients received 56 cycles of treatment. The median number of courses was two (range 1–6). Five patients completed six courses of treatment, of whom two continued on temozolomide alone (at 1000 mg m^−2^) for a further six cycles.

### Haematological toxicity

#### Stage 1

Mild myelosuppression was the most common toxicity observed, but no patients experienced grade 3/4 toxicity. There was one delay for neutropenia. There were no dose reductions (Table 3

**Table 3:** Stage 1 grade of haematological toxicity

	**0**	**1**	**2**
	**Cycles (patients)**		
*Carboplatin AUC 3*			
Anaemia	6 (3)	2(1)	2 (1)
Thrombocytopenia	10 (3)	0(0)	0 (0)
Neutropenia	9 (3)	1(1)	0 (0)
			
*Carboplatin AUC 4*			
Anaemia	4 (3)	6 (3)	1 (1)
Thrombocytopenia	4 (3)	6 (3)	1 (2)
Neutropenia	6 (3)	5 (3)	0 (0)
			
*Carboplatin AUC 5*			
Anaemia	5 (3)	2 (2)	0 (0)
Thrombocytopenia	5 (3)	2 (1)	0 (0)
Neutropenia	5 (3)	2 (1)	0 (0)
			
*Carboplatin AUC 6*			
Anaemia	4 (2)	0 (0)	1 (1)
Thrombocytopenia	4 (3)	1 (1)	0 (0)
Neutropenia	4 (3)	1 (1)	0 (0)

).

#### Stage 2

In stage 2, in all cohorts, 53 cycles of treatment were evaluable for toxicity. No patients experienced significant haematological toxicity at a dose of carboplatin AUC 3. At a carboplatin dose of AUC 4, myelosuppression became more common and significant. The first patient at AUC 4 tolerated two cycles of treatment without significant toxicity. The second patient experienced grade 4 neutropenia and thrombocytopenia with the first cycle of treatment, but recovered to receive the second cycle after the required interval. The third patient also experienced grade 4 neutropenia and thrombocytopenia. However, as the first two patients had tolerated the treatment with minimal subjective toxicity and recovered to receive further treatment on schedule, the dose was then escalated to AUC 5 in accordance with the protocol. Dose escalation should have stopped after two patients experienced DLT, but this only became apparent after the first patient at AUC 5 had tolerated that dose well, which in retrospect led us to treat three further patients at AUC 5 incorrectly.

At AUC 5, the first patient completed two cycles of treatment without significant haematological toxicity. The second patient died on day 15 of treatment. This patient had multiple sites of disease at diagnosis, including liver, splenic and lung metastases, and was taking nonsteroidal analgesia for back pain from bone metastases. On day 6 after the first cycle of treatment, analgesia was increased because of worsening back pain, and on day 15 the patient had a suspected intra-abdominal catastrophe after an episode of vomiting. There was no obvious haematemesis or melaena, so it is unknown whether this was a bleeding complication. The patient did not have a full blood count (it was due that day), so it is unknown whether she was myelosuppressed at the time of death. A postmortem was refused. This was thus recorded as an early death, although it is unknown to what extent chemotherapy contributed to her death.

Two further patients were treated at this dose level, one of whom experienced grade 3 haematological toxicity after the second cycle of treatment, and the other grade 4 haematological toxicity. There were no infectious complications; however, the fourth patient treated at this level required blood transfusions after cycles 1, 2, and 3, and a platelet transfusion after cycle 4 for an episode of epistaxis. Imaging at this time demonstrated a partial response. The patient, however, was considered not to be fit for further chemotherapy due to general debilitation and the response was not sustained. The DLT was thus haematological at carboplatin AUC 5 and further patients were treated at carboplatin AUC 4 to gain further information about tolerability.

In the total cohort of 11 patients treated at carboplatin AUC 4, haematological toxicity remained significant (Table 4

**Table 4:** Stage 2 grade of haematological toxicity

	**0**	**1**	**2**	**3**	**4**
	**Cycles (patients)**				
*Carboplatin AUC 3*					
Anaemia	2 (1)	10 (3)	2 (2)	0 (0)	0 (0)
Thrombocytopenia	7 (3)	2 (1)	5 (2)	0 (0)	0 (0)
Neutropenia	11 (3)	3 (2)	0 (0)	0 (0)	0 (0)
					
*Carboplatin AUC 4*					
Anaemia	20 (9)	6 (4)	3 (3)	2 (1)	1 (1)
Thrombocytopenia	13 (4)	0 (0)	0 (0)	3 (2)	16 (6)
Neutropenia	17 (5)	2 (2)	7 (2)	3 (2)	3 (2)
					
*Carboplatin AUC 5*					
Anaemia	4 (3)	1 (1)	1 (1)	1 (1)	0 (0)
Thrombocytopenia	2 (1)	0 (0)	0 (0)	3 (2)	2 (1)
Neutropenia	3 (2)	1 (1)	0 (0)	2 (2)	1 (1)

). Thrombocytopenia was a frequent toxicity, with six patients experiencing grade 4 toxicity, in 16 of 32 cycles administered. The platelet nadir in all cases was at day 21, but was generally transient and there was only one episode of epistaxis for which the patient received platelet support. The majority of patients recovered their platelet count in time for the next treatment cycle; however, five cycles were delayed because of thrombocytopenia. The dose of carboplatin was reduced in two patients. One patient required a 2-week treatment delay after the first cycle of treatment, and the dose of carboplatin was reduced to AUC 3. Despite this, further thrombocytopenia causing treatment delay was observed and the carboplatin dose was reduced to AUC 2 for the fourth cycle. Grade 4 thrombocytopenia occurred once again and due to ongoing myelosuppression treatment was stopped. The second patient experienced significant anaemia and thrombocytopenia, and the carboplatin dose was reduced to AUC 3 for the sixth cycle of treatment after a 2-week treatment delay for neutropenia.

Neutropenia was observed less frequently. However, prolonged neutropenia resulted in treatment delay in five cycles, and dose reduction was required in two patients. Furthermore, two patients experienced grade 4 toxicity, in one case resulting in neutropenic sepsis requiring hospital admission. This patient died of probable cardiac failure while in hospital following treatment for neutropenic sepsis, although bone marrow recovery had occurred at the time of death. Prolonged neutropenia resulted in treatment delay in five cycles, and dose reduction was required in two patients.

With expansion of the AUC 4 cohort, grade 3/4 myelosuppression remained significant and common, and was thus not found to be the maximum tolerated dose.

### Nonhaematological toxicities

Nonhaematological toxicity was mild, but included gastrointestinal toxicity, with grade 1 nausea and vomiting in six patients, grade 2 in two patients and grade 3 in three patients. Six patients also experienced grade 1 constipation and one grade 3. Five patients experienced grade 1 mucositis. There was no renal or hepatic toxicity.

### Response

#### Stage 1

No patients in phase 1 responded to the treatment combination, athough three patients achieved transient stabilisation of their disease. In all, 12 patients progressed on treatment.

#### Stage 2

Two of 18 patients were not assessable for response due to early death or deterioration. In the whole cohort, two patients achieved a partial response to treatment, one patient in cohort 1 (carboplatin AUC 3) and one patient in cohort 2 at carboplatin AUC 4. Both patients had multiple sites of disease, including lung and soft tissue, and one patient also had liver involvement. Three patients achieved disease stabilisation, and the other 11 patients progressed on treatment. The median duration of response in the two patients achieving a partial response was 7.2 months.

The median overall survival in this group of patients was 5.6 months.

Two patients with stable disease after six cycles of the combination received six further cycles of temozolomide alone. The treatment was well tolerated in both patients, and no progression of disease was seen while they remained on treatment. One patient progressed 4 months after the completion of treatment and the other 11 months later.

## DISCUSSION

Chemotherapy for patients with advanced melanoma is largely palliative with short survival times, thus outpatient regimens with low toxicity are preferable. Temozolomide is an attractive agent for use in melanoma, as it has excellent oral bioavailability, it penetrates the CSF and has the same active component as DTIC, the standard therapy for melanoma. In the Cancer Research Campaign (CRC) phase II trial of temozolomide in melanoma, the results were promising, with an overall response rate of 21% in 49 patients assessable for response. Most patients had multiple sites of metastases. All patients initially received 750 mg m^−2^ given over 5 days, and if no grade 3 or 4 toxicity was observed, subsequent courses were administered at 1000 mg m^−2^ again given over 5 days. In 43 of 55 patients, dose escalation was possible and only two episodes of grade 4 thrombocytopenia and five episodes of grade 4 neutropenia were observed. Tolerability was confirmed in a large phase III trial comparing temozolomide with DTIC, where only 64 of 581 cycles (11%) resulted in grade 3 or 4 neutropenia or thrombocytopenia. In all, 3% of patients discontinued treatment due to adverse events.

Here, when carboplatin (AUC 3–6) was added to temozolomide at 750 mg m^−2^, the regimen was well tolerated with no grade 3 or 4 toxicity. However, when the temozolomide dose was increased to the recommended phase II dose of 1000 mg m^−2^ myelosuppression became more common and significant. The DLT was haematological at carboplatin AUC 5 with two of four patients experiencing grade 3/4 thrombocytopenia and neutropenia. At this dose, there was also an early death on day 15 of the first treatment cycle. Eight patients, in addition to the initial cohort of three patients, were treated at a carboplatin dose of AUC 4 to further assess toxicity. This demonstrated persistent significant myelosuppression with 16 of 32 cycles (50%), resulting in grade 4 thrombocytopenia. In addition, four patients experienced grade 3 or 4 neutropenia, which resulted in neutropenic sepsis requiring hospitalisation admission in one patient. Two patients discontinued treatment due to toxicity, one due to ongoing myelosuppression despite two dose reductions and the other due to general debility after four cycles. Owing to the significance of the toxicity in the expanded cohort at carboplatin AUC 4, this was not the MTD.

Although response was not a primary end point, the response rate was poor in both phases of the trial. At 750 mg m^−2^ temozolomide no patients responded to treatment, although three out 12 achieved disease stabilisation. At 1000 mg m^−2^ temozolomide, two of 18 patients achieved a partial response, and a further three achieved disease stabilisation. Thus, the overall response rate was two of 28 (7%). There were no responses noted in any of the 11 patients with brain metastases. This appears inferior to results from the initial phase I trial of temozolomide that included 23 patients with metastatic melanoma (Newlands *et al*, 1992). There, four patients (17%) responded to temozolomide, two at 750 mg m^−2^ and two at 1000 mg m^−2^ using the same 5-day scheduling. In general, higher response rates have been observed in subcutaneous and lymph node disease, although in the CRC phase II study of temozolomide as a single agent there were three complete responses in patients with lung metastases, and partial responses noted in another nine patients with miscellaneous sites of disease. There were no responses in the brain metastases of four patients. The disease mix here was similar to that in the CRC phase II trial, with 71% patients in our trial having multiple sites of disease, compared to 80% in the CRC trial. However, there was a much higher patient number with brain metastases in the present study (37%). It is disappointing that no responses occurred in our study in patients with cerebral metastases as temozolomide is known to cross the blood–brain barrier, and responses are found in primary brain tumours. In the larger phase III trial where 156 patients received temozolomide, the overall response rate was 13.5%. It was not stated how many patients had multiple sites of metastases, but patients with cerebral metastases were excluded from the trial. A recent phase II trial examined a combination of docetaxel and temozolomide 1000 mg m^−2^ given 4-weekly in 62 patients with metastatic melanoma (Bafaloukos *et al*, 2002). Again the treatment was well tolerated with only eight patients (13%) experiencing grade 3/4 thrombocytopenia, and an overall response rate of 27% was achieved. Three of eight patients with brain metastases responded to treatment.

Despite the poor response rate of the combination of treatment in the present study, the median survival in this group of patients was 5.6 months, which was in keeping with that of treatment with other chemotherapy agents.

In conclusion, the addition of carboplatin to temozolomide 1000 mg m^−2^ significantly adds to myelosuppression when the carboplatin dose is escalated over AUC 3. Grade 4 thrombocytopenia is common at day 21 with carboplatin AUC 4, although the regimen is otherwise well tolerated. In this population of patients, with a high proportion of brain metastases, the response rates were low (7%) and less than previously reported. Owing to poor response rates, it was not thought justifiable to expand the cohort of patients receiving a carboplatin dose of AUC 3, and thus the MTD was not defined. It is unlikely that the combination of temozolomide and carboplatin can be given in sufficient doses to improve on the efficacy of temozolomide given as a single agent in advanced melanoma.
